# Practical Anatomy of the Nerves and Vessels Supplying the Head, Neck, and Chest: Intended as a Guide for the Use of Students in the Dissection of Those Structures

**Published:** 1836-04

**Authors:** 


Art. XVIII.—*Practical Anatomy of the Nerves and Vessels supplying the Head, Neck, and Chest: intended as a Guide for the Use of Students in the Dissection of those Structures.* By E. Cock, Demonstrator of Anatomy at Guy's Hospital.—*London*, 1835. 12mo. pp. 242.

This publication bears alike in its plan, in its descriptions, and in the extent to which these have been carried, the marks of an author practically acquainted with the subject of anatomical demonstration, and must prove a valuable assistant of the student in the manual part of his anatomical studies, for which it is exclusively intended. Those who have experienced the inconvenience of examining regions of the body, (for descriptions of the parts contained in which they have been obliged to refer to different pages of indifferently indexed anatomical works,) will fully appreciate the facility which is afforded to the study of dissection, by a book which guides them from the external to the deeply-seated parts, with descriptions confined to those portions of vessels and nerves, with their relations, which come successively into view; and although it is not unfrequently a necessary consequence of the plan Mr. Cock has followed, that small portions of such vessels and nerves can alone be exposed at the same time, and a frequent recurrence to the same in another part of their course is consequently required, still this objection is fully answered by the author, by the remark, that the student, in the descriptive method generally followed, finds the part which he has been dissecting the reverse of that which he has so carefully studied in his book; and that, in order to accommodate the one to the other, he must begin at the end of the chapters, and read backwards.

Another recommendation of the order here adopted is, that "every important part contained in the head, neck, and chest, may be seen during the dissection of a single subject;" a point which the young student would find it very difficult to ensure by following the directions of any book of anatomical demonstration with which we are acquainted. A knowledge of the bones, muscles, and viscera, in relation with, and supplied by, the nerves and vessels which are described, is required previous to the use of the present work. Our chief object will be to illustrate the plan; as of the anatomy, considered apart from its arrangement, little will require to be said.

We will suppose the skin of one side of the neck and face, with the platysma, to be removed; the parts exposed are the superficial branches of the cervical plexus; the termination of the accessory nerve; the external jugular vein; above, the ramifications of the portio dura and the external terminations of the fifth pair. The removal of the cervical fascia exposes in front the anterior triangular space of the neck, with its contents; part of the lingual nerve with its descending offset; another portion of the accessory nerve; the facial vein, the sheath of the carotid and its contents; the origin and course of its branches through the triangle, and such of them to their terminations as may be followed without interfering with the regular course of the dissection. Thus, for example, the lingual, occipital, ascending pharyngeal and internal maxillary arteries, are traced through but a small portion of their course; while the description of the superior thyroid, facial, sterno-mastoid, posterior aural and temporal, is almost completed. The next step of the dissection (and the best method of exposing the parts is very well described,) is the examination of the remaining course of the lingual artery and nerve with the gustatory nerve; and, subsequently, the exposure of the internal maxillary artery, with the superior and inferior maxillary nerves. The removal of the lower jaw and muscles connected with the styloid process exposes the ascending pharyngeal artery, the internal carotid and jugular vein, with the nerves emerging at that part from the base of the skull, the terminations of some of which have already been traced. The remainder of the occipital artery is necessarily left to a subsequent stage of the dissection.

It is unnecessary any farther to illustrate the plan which has been followed, in the same manner and with equal clearness, with regard to all the vessels and nerves of the head, neck, and chest. The anatomical descriptions are all concisely and clearly written.

There is one point into which the author might perhaps have occasionally entered with advantage rather more into detail,—the frequent varieties in the origins of arteries. Students are occasionally much at fault, on finding exceptions in the subject which they are dissecting to the standard rule; and a specification of these exceptions is often a great assistance. Thus, in describing the thyroid axis, as most generally the source of four branches, and qualifying this by a statement that there are many exceptions to this rule, a few lines might have been judiciously added, specifying those exceptions. It might be considered hypercriticism to apply a similar remark to the distribution of the ophthalmic artery, but the occasional origin of a vessel supplying the parts within the orbit, from other than the ophthalmic artery, and the variations in the source of the branches generally derived from the main trunk, but which sometimes originate in its secondary branches, sometimes perplex a young student in that dissection. We prefer Cloquet's division of the internal maxillary artery to that adopted by Mr. Cock; as, without extending the limits of the spheno-maxillary fossa beyond those which are properly assigned to it, this vessel can scarcely be said to detach from that part the branches described. Mr. Cock appears to have been indebted to his own observation for his anatomy, and it is perhaps but a proof of the want of uniformity in the distribution of vessels, that he has spoken of the facial artery as passing through the substance, instead of on the surface of the submaxillary gland, or that he has mentioned but one occipital sinus. It can, however, scarcely be correct to speak of the longitudinal sinus as opening into the right and left lateral sinuses, as the torcular herophili is an intermediate receptacle between these vessels.

The facial artery is also mentioned as lying between the masseter and levator, instead of the depressor anguli oris; and the carotid artery within the skull as situated external to the ophthalmic nerve.

The departure from the common numerical arrangement of the cerebral nerves, describing as distinct pairs what are commonly termed the seventh; and, instead of including the par vagum, glosso-pharyngeal and spinal accessory nerves as the eighth pair, separating the first two, as the ninth and tenth, and the third, as no cerebral nerve at all, is certainly more consistent with anatomical accuracy.

